# Efficacy of a Digital Postoperative Rehabilitation Intervention in Patients With Primary Liver Cancer: Randomized Controlled Trial

**DOI:** 10.2196/59228

**Published:** 2025-04-07

**Authors:** Kaitao Yu, Baobing Yin, Ying Zhu, Hongdao Meng, Wenwei Zhu, Lu Lu, Junqiao Wang, Shugeng Chen, Jun Ni, Yifang Lin, Jie Jia

**Affiliations:** 1 Department of Rehabilitation Medicine, Huashan Hospital, Fudan University, Shanghai, 200040, China, 86 136 1172 2357; 2 Department of General Surgery, Hepatobiliary Surgery, Huashan Hospital, Fudan University, Shanghai, China; 3 Department of Hepatobiliary Surgery, National Regional Medical Center, Binhai Campus of the First Affiliated Hospital, Fujian Medical University, Fuzhou, China; 4 College of Behavioral & Community Sciences, School of Aging Studies, University of South Florida, Florida, FL, United States; 5 School of Nursing, Fudan University, Shanghai, China; 6 Department of Rehabilitation Medicine, National Regional Medical Center, Binhai Campus of the First Affiliated Hospital, Fujian Medical University, Fuzhou, China

**Keywords:** digital health, surgery, exercise rehabilitation, randomized controlled trial, primary liver cancer

## Abstract

**Background:**

Rehabilitation is considered a fundamental component of cancer treatment, especially for patients undergoing cancer surgery. In contrast to conventional rehabilitation education, digital rehabilitation has the potential to improve patients’ access to postoperative rehabilitation programs. While digital health has rapidly emerged to aid patients with various diseases, their clinical efficacy in the recovery of patients with primary liver cancer (PLC) undergoing hepatectomy remains inadequately investigated.

**Objective:**

This study aims to evaluate whether a digital postoperative rehabilitation intervention is efficient in improving physical fitness, enhancing exercise adherence, and alleviating fatigue among patients with PLC after hepatectomy.

**Methods:**

A randomized controlled trial was undertaken across 2 university-affiliated hospitals in Eastern China. A total of 100 participants were enrolled in this study and were allocated randomly to either the digital health (intervention group, n=50) or the rehabilitation manual-based group (control group, n=50) at a 1:1 ratio. Patients were unblinded and prospectively followed for the intervention of 3 weeks. Outcome measures included physical fitness, exercise adherence, and status of fatigue.

**Results:**

Overall, 91 out of 100 patients completed the research and were evaluated after 3 weeks of intervention. The digital health group showed better cardiopulmonary endurance than the control group. The mean difference in the change of 6-minute walk test distance from baseline between the groups was 70.21 (95% CI 0.730-82.869) m (*P*=.05). No statistically significant effects were found for grip strength, 5-repetition-sit-to-stand test time, and fatigue. The exercise adherence in the digital health group was higher than that in the control group (*χ*^2^_2_=15.871, *P*<.001).

**Conclusions:**

The findings suggested that the implementation of digital health had a positive impact on recovery in exercise capacity after hepatectomy. In addition, rehabilitation exercise mode based on digital health has the potential to improve the exercise adherence of patients with PLC compared to conventional manual-based rehabilitation guidance.

## Introduction

Primary liver cancers (PLCs), including hepatocellular carcinoma and intrahepatic cholangiocarcinoma, are associated with significant morbidity and mortality [1]. Over the years, advanced hepatic surgical techniques have led to the development of safe and effective therapies for patients with PLC. Nonetheless, the complex procedure of hepatic resection results in a decline in physical fitness and a high incidence of postoperative adverse physical symptoms such as fatigue, which hampers quality of life and disrupts daily activities [2,3].

Increasing evidence suggests that a combination of multimodal exercise interventions can improve physical fitness and function for patients with PLC after hepatectomy [4]. In addition, enhanced recovery after surgery is mostly confined to the perioperative period. Patients face problems in self-management after discharge and thus have difficulty in exercise adherence [5], which is the extent to which individuals undertake prescribed behavior accurately and at the agreed frequency, intensity, and duration [6]. Physical inactivity has been described as the greatest public health threat. It is estimated that as few as 26% of men and 19% of women adhere to the guidelines [7]. Despite this, patients with PLC may also feel ambiguous about the content of rehabilitation and have difficulty understanding how to balance exercise and discomfort due to the complexity of surgical incisions and side effects [8]. In patients undergoing hepatectomy, the type, intensity, and amount of exercise rehabilitation must be adapted to their individual performance levels [9]. Therefore, advanced interventions of rehabilitation and real-time feedback are important to increase exercise adherence, which may eventually contribute to improving physical fitness and reducing adverse symptoms. In 2021, over 1.028 billion Chinese individuals, accounting for 73% of the Chinese population, accessed the internet via mobile phones. Digital health is an important medium for the delivery of rehabilitation interventions based on mobile devices, providing health education to improve the awareness of liver cancer rehabilitation, assisting in self-management and improving compliance with liver cancer rehabilitation, real-time exercise monitoring to improve the safety of discharged liver cancer rehabilitation, which could serve as a viable and accessible support platform. It significantly enhances outreach to the extensive oncological patient population [10].

There have been no studies that examined the clinical efficacy of digital health-based exercise programs for patients with PLC after hepatectomy during the discharge transition period in China. To measure the impact of exercise rehabilitation based on digital health, we hypothesized that exercise rehabilitation supervised through digital health would be more efficient than traditional unsupervised interventions like manual-based exercise education in improving physical outcomes for patients with PLC.

## Methods

### Study Design

A prospective, randomized controlled trial was conducted in 2 university-affiliated hospitals (Huashan Hospital of Fudan University in Shanghai and The First Hospital of Fujian Medical University Hospital in Fujian, China). Randomization was 1:1 into the digital health group (intervention group) or the rehabilitation manual-based group (control group). They were requested to complete 2 circles of assessments with baseline as the first time before surgery and subsequent assessments after 3 weeks’ intervention.

### Study Participants

This study was intended for patients with PLC after hepatectomy. Participants aged 35 to 75 years who met the following inclusion criteria were approached to participate: (1) patients with PLC expected to undergo hepatectomy 2‐4 liver segments, progressive PLC (stage 2 or 3) for open or laparoscopic hepatectomy; (2) Child-Pugh A; (3) patients with an oncology history of PLC that has not recurred within 3 years after surgery; and (4) ability to operate a mobile phone. Notably, patients were excluded if they had exercise, psychological, or cognitive disorders before hepatectomy.

Appropriate participants were jointly identified by the chief rehabilitation therapists and chief surgeons. Participants were given ample time to consider and voluntarily choose to participate in the study, with written informed consent obtained the day before surgery. Before obtaining consent, researchers thoroughly explained the research procedures and detailed the preoperative and follow-up data to be collected in the ward. The methods and specific data content were clearly outlined in the informed consent form. All participants received standard postoperative care in the operating room, postanesthesia care unit, and ward.

### Intervention

The R Plus Health app (Recovery Plus), a medical device cleared by the Chinese FDA, served as the underlying software and hardware for the intervention in this trial, including the basic app structure and the target heart rate belt to guide and supervise training exercises. Based on this, a 3-week exercise program specifically designed for patients with PLC after hepatectomy was developed by an oncology exercise rehabilitation team, which consisted of 3 hepatobiliary surgical oncologists, 2 rehabilitation doctors, 2 nurses, and 3 physiotherapists. After the preliminary literature review and expert demonstration meeting, a set of exercise rehabilitation plans for patients with PLC surgery after discharge was discussed [11]. Reference to the framework in the guide for "FITT Principle” (Exercise prescriptions include the 4 elements of exercise frequency, intensity, time, and type), aerobic exercise, resistance exercise, and stretching training are in the exercise type. The exercise frequency was 30‐50 minutes each time, 3‐5 times per week, and 90‐150 minutes each week. The oncology rehabilitation program consists of 4 parts: warm-up movements, whole-body exercises, stretching exercises, and finishing exercises. Exercise intensity is recommended to choose low-intensity movements during the warm-up and finishing stages, and moderate intensity during full-body activities. The details of the 4 modules of the oncology rehabilitation program are described in Table 1. This program was subsequently integrated into the app by the software manufacturer.

**Table 1.** Objectives of intervention sessions for exercise rehabilitation.

Stage	Rehabilitation
Warm-up movements	(1）Sitting abdominal breathing: 1 set for 60 s; rest (30 s)(2）Still squat against the wall: 1 set for 60 s; rest (30 s)(3）Step in place: 1 set for 60 s; rest (30 s)(4）Front kick stepping: 1 set for 60 s; rest (30 s)(5）Side leg raises with chair: 1 set for 60 s; rest (30 s)
Stretching sessions	(1) Seated quadriceps stretch: 2 sets for 60 s; rest (30 s)
Whole body sessions	(1）Brisk walking training: 2 sets for 1120 s; rest (30 s)(2）Standing elastic band curls: 2 sets for 160 s; rest (30 s)(3）Micro squats against the wall: 2 sets for 160 s; rest (30 s)(4）Sitting-standing core stabilization: 2 sets for 160 s; rest (30 s)(5）Chair alternating side leg raises: 2 sets for 160 s; rest (30 s)(6）Sitting deltoid stretch (both sides): 2 sets 1120 s; rest (30 s)(7）Seated elastic band shoulder flexion: 2 sets for 160 s; rest (30 s)(8）Brisk walking training: 2 set for 1120 s; rest (30 s)
Finishing sessions	(1）Sitting abdominal breathing: 1 set for 60 s; rest (30 s)(2）Step in place: 1 set for 60 s; rest (30 s)(3）Micro squats against the wall: 1 set for 60 s; rest (30 s)(4）Side leg raises with a chair: 1 set for 60 s; rest (30 s)

### Digital Rehabilitation Group

**Figure 1.**
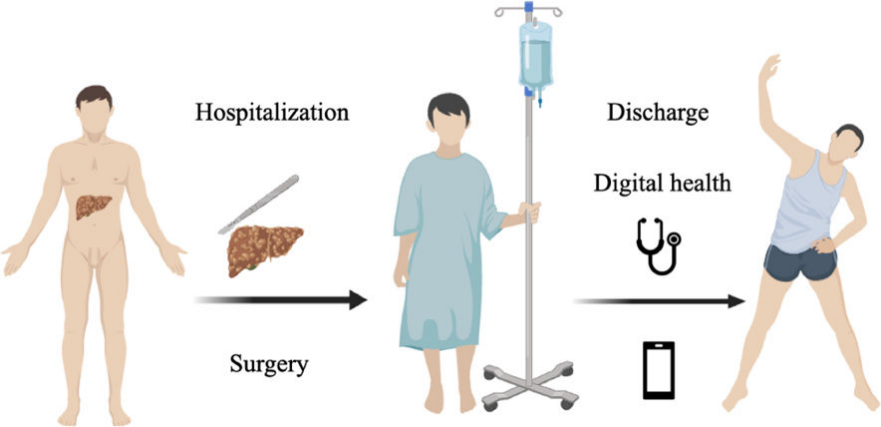
Liver cancer care pathway.

Participants in the intervention group were provided with a QR code to download the app onto their mobile phones (available for both Android and iOS) before discharge. Upon approval by the researchers, participants were wirelessly connected to a chest-worn heart rate monitor, which measured exercise frequency, intensity, duration, and progression and ensured safety. The exercise intensity was performed at 64%‐76% of the age-predicted maximal heart rate (220-age) [12]. When participants first logged into the app, a tutorial guide video was displayed and saved in the tutorial section of their personal account. The exercise prescription was posted to each participant before discharge, teaching them how to follow the video action training. The content of the training was consistent with intervention sessions for exercise rehabilitation (Table 2). Every time when patients finished the rehabilitation prescription, the APP provided the questionnaire scale (Borg Breathing Scale) and the option for the patient to record the discomfort symptoms and feelings, combined with the record of the heart rate during exercise, which was transmitted to the doctor’s platform in time. The doctors adjusted the order and number of movements in the exercise program according to the patient’s information (Figures1  2). In this trial, different patients had varying exercise capacities. Weaker patients could choose to skip movements they were unable to complete, and the system informed the doctor to make appropriate adjustments. Patients initially unable to complete 30‐50 minutes could exercise in smaller increments throughout the day but were encouraged to exercise for 90‐150 minutes per week. The software automatically uploaded the amount of exercise and daily exercise time.

**Table 2.** Baseline characteristics of study participants by treatment.

	Control (N=50)	Digital health (N=50)	Total (N=100)	*P* value
Age (years)				.39
N	50	50	100	
Mean (SD)	56.4 (9.7)	54.6 (10.6)	55.5 (10.2)	
Female, n (%)				.16
0^a^	40 (80.0)	45 (90.0)	85 (85.0)	
1^b^	10 (20.0)	5 (10.0)	15 (15.0)	
BMI (kg/m^2^)				.62
N	50	50	100	
Mean (SD)	24.2 (2.7)	23.9 (3.4)	24.0 (3.1)	
Hypertension, n (%)				.33
0	41 (82.0)	37 (74.0)	78 (78.0)	
1	9 (18.0)	13 (26.0)	22 (22.0)	
Diabetes, n (%)				>.99
0	45 (90.0)	45 (90.0)	90 (90.0)	
1	5 (10.0)	5 (10.0)	10 (10.0)	
HBV positive, n (%)				.66
0	15 (30.0)	13 (26.0)	28 (28.0)	
1	35 (70.0)	37 (74.0)	72 (72.0)	
Tobacco, n (%)				.69
0	21 (42.0)	23 (46.0)	44 (44.0)	
1	29 (58.0)	27 (54.0)	56 (56.0)	
Alcohol, n (%)				.31
0	26 (52.0)	31 (62.0)	57 (57.0)	
1	24 (48.0)	19 (38.0)	43 (43.0)	
Education, n (%)				.33
Illiterate	4 (8.0）	3 (6.0）	7 (7.0)	
Preliminary	16 (32.0）	10 (20.0）	26 (26.0)	
Junior	21 (42.0）	28 (56.0）	49 (49.0)	
Senior	5 (10.0）	2 (4.0）	7 (7.0)	
College or above	4 (8.0）	7 (14.0）	11 (11.0)	
Income (US $/month)，n (%)				.73
<420	20 (20.0）	17 (17.0)	37 (37.0)	
421-840	20 (20.0)	20 (20.0)	40 (40.0)	
>840	10 (10.0)	13 (13.0)	23 (23.0)	
HCC^c^, n (%)				.83
0	15 (30.0)	16 (32.0)	31 (31.0)	
1	35 (70.0)	34 (68.0)	69 (69.0)	
Open surgery, n (%)				.23
0	27 (54.0)	21 (42.0)	48 (48.0)	
1	23 (46.0)	29 (58.0)	52 (52.0)	
Surgery duration (min)				.14
N	50	49	99	
Mean (SD)	147.2 (63.3)	160.9 (53.0)	154.0 (58.5)	
IBL^d^ (mL)				.20
N	50	49	99	
Mean (SD)	297.0 (252.2)	428.1 (471.6)	361.9 (380.9)	
LOS^e^ (days)				.27
N	50	50	100	
Mean (SD)	7.6 (3.4)	8.5 (4.6)	8.1 (4.1)	
Maximum tumor diameter (cm)				.22
N	46	46	92	
Mean (SD)	4.6 (3.6)	5.0 (2.7)	4.8 (3.2)	

a 0=No.

b 1=Yes.

c HCC: hepatocellular carcinoma.

d IBL: intraoperative blood loss.

e LOS: length of stay.

**Figure 2.**
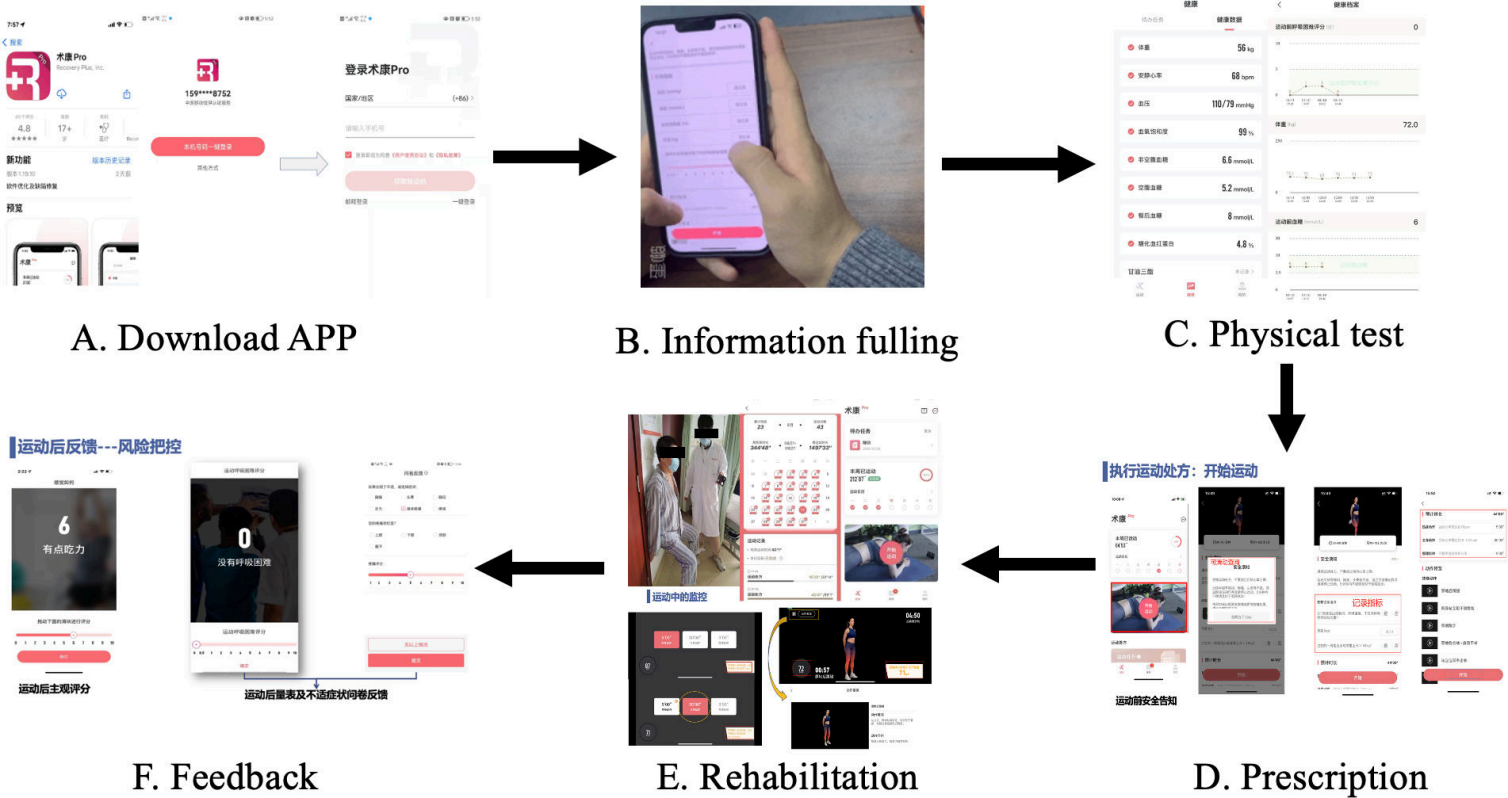
Digital health intervention process.

### Rehabilitation Education Group

From discharge from the hospital to 3 weeks until follow-up, patients received a paper-format rehabilitation manual in exercise rehabilitation, the content of rehabilitation training was consistent with that of the digital health group, and rehabilitation maneuvers were guided before discharge patiently until patients could master them. Patients were asked to write down their daily exercise content in the exercise diary. After discharge, patients were supervised via the web once a week. If there were any problems with the exercise, participants could contact the researchers by phone and receive instructions until the outpatient follow-up visit. Participants were followed up in the outpatient clinic at the end of the 3 weeks after discharge from the hospital.

### Outcome Measures

#### Patient Characteristics

General demographic and clinical characteristics measurements were designed by the research team. Demographic characteristics included age, sex, educational level, and history of diabetes, hypertension, smoking, drinking, and HBV. Clinical characteristics included classification and stage of cancer, surgical procedure, length of hospital stay, and intraoperative blood loss. Demographic characteristics were recorded before surgery, and clinical characteristics were recorded through the electronic medical system one day after surgery.

#### Primary Outcome Measure

The 6-minute walk test (6MWT) was used to evaluate the cardiopulmonary fitness level of the patients with PLC. The test method was to select a 50-meter-long hard surface in the corridor outside the ward and place markers at both ends. Patients were asked to walk back and forth along the marked line as far as possible, ensuring that there was no interference in the surrounding area, talking, running, and jumping were prohibited during the walk, and there was no hesitation at the folding point, and the total distance traveled was calculated after timing the walk for 6 minutes [13]. The 6MWT was selected for its ease of administration, greater tolerability, and superior reflection of cardiopulmonary fitness in patients undergoing abdominal cancer surgery [14].

#### Secondary Outcome Measure

##### Upper Limb Muscle Strength

Grip strength is the sum of the forces produced when holding a target object tightly with the hand in each situation, and its force is produced by the joint contractile activity of the lateral forearm muscle group and the intrinsic hand muscle group [15]. In this study, the Jamar + grip dynamometer was used, which was measured by maintaining a seated position, adjusting the gripping position according to the size of the hand, and grasping the grip dynamometer with force. Each hand was repeated 3 times, and the tester was required to record all the test results, and the maximum value was taken as the final measurement [16]. Patients undergoing major abdominal surgery endure a significant reduction in muscle mass, which exacerbates postoperative outcomes [17-19].

##### Lower Limb Muscle Strength

The Five Times Sit-to-Stand test was used to assess the lower limb muscle strength, balance, and locomotor ability of individuals [20]. This was done by preparing a chair with a height of 48 centimeters, and subjects were instructed to sit with their arms crossed over their chests and their backs against the chair. The sitting and standing test maneuver was repeated 5 times at the fastest speed under the instructor’s command, and the timing was stopped when the subject’s body touched the chair after the 5th time, and the time was recorded [21]. Five Times Sit-to-Stand test is a valid and reliable objective screening tool for muscle strength and frailty of cancer survivors.

##### Fatigue

The fatigue was assessed using the Multidimensional Fatigue Inventory-20, which was developed by Dutch in 1995, contains 20 subjective entries, and was used to assess the severity of fatigue in patients. Higher scores on the scale indicate more severe fatigue. The application of the Chinese version in a cancer population showed a Cronbach α coefficient of 0.867 and good internal consistency [22].

##### Exercise Adherence

There is no standardized scale for exercise adherence. Recommended exercise time is at least 90 minutes per week. In this study, according to the constructed exercise rehabilitation program, sensor- and app-based log files were individually analyzed for each exercise session to quantify exercise adherence. The digital health group was evaluated according to the degree of the patients’ achievement of exercise through the feedback of the exercise record time in the app and the exercise diary of the control group, and the patients’ achievement of exercise amount was evaluated, and the patients’ exercise adherence was regarded to be high if they reached 80% and above of the total recommended exercise time, 40%‐80% was regarded as medium exercise adherence; and below 40% was regarded as low exercise adherence.

##### Sample Size, Randomization, and Blinding

To determine the sample size, we calculated the primary outcome based on 6-minute walking distance in the pretest, we obtained a 6-minute walking distance of 480 (SD 100) m after 3 weeks of intervention for the digital health intervention group and 400 (SD 100) m for the control group. A total of 68 participants was required, calculated with a statistical power of 0.80 and a significance level of .05. Anticipating a 15% dropout rate, the adjusted sample size for our study was set at 80 participants. The web-based research randomizer was used, and a blocked design was selected to ensure balanced allocation across the 2 groups. For each hospital, participants were randomized to the intervention group or the control group to ensure an allocation ratio of 1:1.

All eligible patients provided informed consent according to the protocol and agreed to participate in the study. They were then centrally randomized into either the digital health group (intervention group) or the manual-based group (control group) at a 1:1 ratio. The randomization code was generated using a computer random number generator by researchers who were not involved in patient treatment. Stratified randomization was conducted by performing the randomization procedure separately for each of the 2 centers. Participants were not blinded to group assignment or type of study intervention, but study personnel responsible for data collection and analysis were blinded.

### Data Collection

Data were collected between January 2022 and October 2023. After signing the paper consent form, all participants completed the demographic and baseline physical outcomes before randomization (T0). Participants in both groups also completed the assessment at 3 weeks (T1) when they followed up. If participants did not return to the hospital, researchers contacted them by phone to collect related information.

### Statistical Methods

Data were analyzed using IBM SPSS (version 26.0; IBM Corp) and Stata BE (version 17; Stata Corp). Primary and secondary outcomes were analyzed as follows: using the Kolmogorov-Smirnov test to determine whether continuous data follows a normal distribution. Count data were presented as frequencies and percentages (%). Continuous data following normal distribution were expressed as mean and SD, while nonnormal distributions were expressed as median (quartile spacing). Because the outcome variables were measured twice before and after intervention, a difference in differences approach (linear regression) was used to control for baseline levels. The independent samples *t* test was used for comparison between groups of normally distributed continuous data. The Mann-Whitney *U* test was used for comparison of nonnormally distributed continuous data. Count data were expressed as the number of cases (rate) and tested using the chi-square test or Fisher exact test. Mean differences in the change from baseline to week 3 were also calculated. If subsequent multi-factor analysis is required, multiple linear regression or logistic regression models are selected according to continuous-type or subtype dependent variables. Multiple interpolations were used for the analysis of missing data. *P*<.05 was considered significant.

The study used mixed-effects linear regression models to examine the preliminary impact of the intervention while adjusting baseline characteristics. The model incorporated fixed effects for age, sex, education levels, hypertension status, drinking status, and time. To account for the hierarchical structure of the data (patients nested within 2 hospitals), the model included random intercept and slope at the hospital level. At the patient level, random slope and intercept for a time were included to allow variations between individual patients in terms of initial status and trajectories over time on the outcome variables. The model is estimated using maximum likelihood estimation [23,24].

### Ethical Considerations

This study was approved by the institutional review board of Huashan Hospital, Fudan University (approval number 2021-794). The research procedures were conducted in accordance with the ethical standards of the responsible committee on human experimentation (institutional and national) and the World Medical Association Declaration of Helsinki. All participants provided written informed consent prior to their participation in the study. To ensure privacy and confidentiality, all collected data were anonymized and securely stored. Access to the study application was protected by individual private usernames and passwords assigned to each participant. No financial compensation was provided to participants for their involvement in the study, and all study materials were provided free of charge. The study was registered at ClinicalTrials.gov (Identifier: ChiCTR2100052911).

## Results

### Participant Timeline

Of the 254 patients screened, 99 patients did not meet the inclusion criteria. Specifically, 72 patients were diagnosed with benign liver tumors, 17 patients were expected to undergo hepatectomy involving fewer than 2 liver segments, and 10 patients were unable to operate a mobile phone. Additionally, 28 patients declined to participate without specifying reasons, 6 patients were not enrolled due to lack of family support, 2 patients refused because they were uncertain about the trial’s usefulness, 12 patients lacked interest, and 7 patients declined for other unspecified reasons.

Of the 100 patients who met the criteria, 50 patients were randomly assigned to the digital health group (n=45, 90% male patients; the mean age was 54.6, SD 10.6 years) and 50 to the control group (n=40, 80%) male patients; the mean age was 56.4, SD 9.7 years). At follow-up, 5 patients in the digital health group were lost: 1 patient lost contact, 1 patient cited time constraints, 1 patient withdrew voluntarily, and 2 patients withdrew due to an allergic reaction to the heart rate monitor. In the control group, 4 patients were lost to follow-up: 2 patients lost contacts, 1 patient died, and 1 patient withdrew voluntarily.

**Figure 3.**
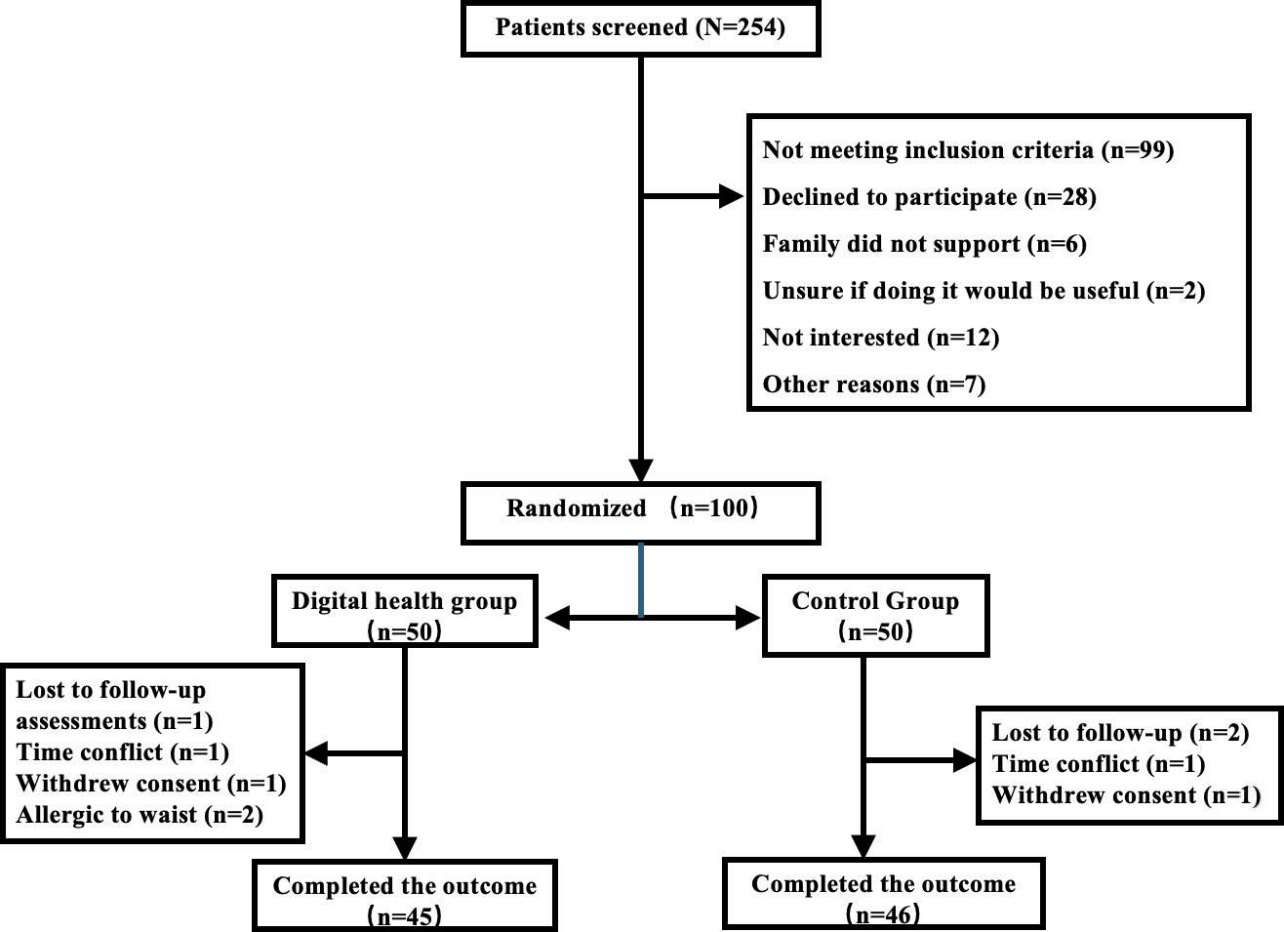
Participant timeline.

A total of 91 participants completed all assessments, with a dropout rate of 10% (5/50) in the digital health group and 8% (4/50) in the control group. No significant differences were detected between those who were lost to follow-up and those who completed the assessments, as none of the *P* values reached statistical significance. For more details, refer to Figure 3 for the participant timeline.

### Baseline Data

Participants’ ages ranged from 35 to 75 (average 55.5, SD 10.2) years. No significant differences were detected in the baseline characteristics between the digital health and control groups (*P*>.05). The majority (69/100, 69%) of the patients were diagnosed with hepatocellular carcinoma. No significant differences were detected in the surgery characteristics between the digital health and control groups (*P*>.05). Details are listed in Table 2.

### Primary Outcome

The 6MWT demonstrated a significant improvement in the intervention group compared with the control group. At baseline, the mean 6MWT distance was 515.3 (SD 127.6) m in the intervention group and 520.0 (SD 73.7) m in the control group. After 3 weeks, the mean 6MWT distance in the intervention group decreased to 501.0 (SD 114.1) m, whereas the control group showed a more pronounced decline to 464.0 (SD 100.7) m. The mean difference in the change from baseline between the groups was 70.21 (95% CI 0.730-82.869) m (*P*=.05), indicating a statistically significant improvement in the intervention group over the control group.

### Secondary Outcomes

#### Grip Strength

The mean grip strength at baseline was comparable between groups, with the intervention group showing 34.8 (SD 9.3) kg and the control group 33.4 (SD 9.8) kg. Posttreatment, the intervention group had a slight improvement to 34.9 (SD 9.0) kg, while the control group decreased to 31.8 (SD 8.6) kg. The mean difference between the groups was 1.76 (95% CI −0.288 to 3.814) kg. However, this difference was not statistically significant (*P*=.09).

#### 5-Repetition Sit-to-Stand Test

Baseline scores for the 5-repetition-sit-to-stand test were 9.2 (SD 3.0) seconds in the intervention group and 9.7 (SD 4.7) seconds in the control group. Posttreatment, the intervention group improved to 8.9 (SD 2.4) seconds, whereas the control group worsened slightly to 10.0 (SD 3.1) seconds. The mean difference between groups was 0.62 (95% CI −2.236 to 0.934) seconds, but the result was not statistically significant (*P*=.42).

#### Fatigue Score (Multidimensional Fatigue Inventory-20)

Fatigue scores were measured at baseline and posttreatment. The intervention group showed a mean baseline fatigue score of 44.8 (SD 9.8) points, identical to the control group (mean 44.8, SD 9.8). However, after treatment, the intervention group’s fatigue score increased to 47.4 (SD 10.8) points, while the control group saw a more substantial increase to 55.9 (SD 11.6) points. The mean difference in the change from baseline was −2.77 (95% CI −7.919 to 1.271) points, which was not statistically significant (*P*=.16). Details are listed in Table 3.

**Table 3.** Comparison of physical function between 2 groups.

Change from baseline at week 3	Pretreatment				Posttreatment				Mean difference from baseline to week 3, (95% CI)	*P* values
	Patients number	Intervention, mean (SD)	Patients number	Control, mean (SD)	Patients number	Intervention, mean (SD)	Patients number	Control, mean (SD)		
6MWT^a^ (meters)	47	515.3 (127.6)	49	520.0 (73.7)	47	501.0 (114.1)	49	464.0 (100.7)	70.21 (0.730 to 82.869)	<.05
Grip (Kg)	46	34.8 (9.3)	49	33.4 (9.8)	46	34.9 (9.0)	49	31.8 (8.6)	1.76 (−0.288 to 3.814)	.09
5R-STS^b^ (s)	48	9.2 (3.0)	48	9.7 (4.7)	48	8.9 (2.4)	48	10.0 (3.1)	0.62 (−2.236 to 0.934)	.42
Fatigue score (points)	50	44.8 (9.8)	49	44.8 (9.8)	49	47.4 (10.8)	49	55.9 (11.6)	−2.77 (−7.919 to 1.271)	.16

a 6MWT: 6-minute walk test.

b 5R-STS 5-repetition-sit-to-stand test.

### Intervention Adherence and Completion

The chi-square test was used to compare the exercise compliance between the 2 groups, and the results showed that compared with the control group, the exercise compliance of the experimental group was significantly higher than that of the control group at 3 weeks after discharge (*χ*^2^_2_=15.87, *P*<.001), and the difference was statistically significant, as shown in Table 4.

**Table 4.** Comparison of exercise adherence between 2 groups.

Adherence	Control group (n=50)	Digital health (n=50)	Chi-square (*df*)	*P* value
High adherence, n (%)	6 (12.0）	18 (36.0）	15.87 (2)	<.001
Medium adherence, n (%)	17 (34.0）	3 (6.0）	—^a^	—
Low adherence, n (%)	27 (54.0）	29 (58.0）	—	—

a Not available.

## Discussion

### Overview

In recent years, rapid advancements in the treatment of PLC have significantly improved overall survival rates for patients [25]. Rehabilitation, increasingly recognized as a vital component of postoperative care, is delivered through a multidisciplinary team. The benefits of exercise are well-documented by authoritative bodies [26]. However, exercise rehabilitation remains a valuable yet underused treatment strategy for patients with PLC. In this trial, the digital health rehabilitation program was rigorously validated by a multidisciplinary team and specifically designed for patients with PLC posthepatectomy in China. This pilot study aimed to compare the efficacy of a digital health intervention with a conventional rehabilitation manual on physical fitness and symptoms in patients with PLC following hepatectomy over a 3-week intervention period. The findings demonstrated a statistically significant improvement in cardiopulmonary fitness (as measured by the 6-minute walk distance) in the digital health group compared with the control group. However, no statistically significant effects were observed for secondary outcomes. Importantly, exercise adherence was significantly higher in the digital health group.

### Principal Findings

#### Performance Measures

The results showed that the digital health program significantly improved the cardiopulmonary fitness (primary outcome) of patients after hepatobiliary surgery at 3 weeks. The comparison between groups’ mean differences value in 6-minute walking distance was 70.21 (95% CI 0.730-82.869) m (*P*=.05). Although the minimal clinically important difference for 6MWD in patients with PLC after hepatectomy remains undetermined. In contrast to previous research indicating that home-based exercise or telerehabilitation interventions in patients with cancer have demonstrated only minor to moderate effects on 6-minute walking distance or oxygen uptake [27,28]. The improvement in this trial is in the range of the conservative estimate of minimal clinically important difference, indicating a statistically significant improvement in the digital health group [29]. Significant changes in cardiorespiratory performance resulted in an inverse and clinically relevant change in mortality risk and contributed to improved health [30]. Golbus et al [31] and Kim et al [32] also validated the statistically significant improvements in 6MWD using a digital health app.

Regarding secondary outcomes, there were no statistically significant differences between the 2 groups in terms of upper and lower limb muscle strength and fatigue. Although the digital health group exhibited a slight increase in grip strength and a decrease in the time required to complete the five sit-to-stand tests compared with the control group, these changes did not reach statistical significance. However, considering that interventions such as quadriceps stretching, seated deltoid stretches, elastic band exercises, and wall squats are designed to enhance overall muscle mass, improvements in muscle strength and fatigue over time may hold clinical significance.

#### Adherence

In this trial, the digital health program significantly increased exercise adherence compared with the control group (*P*<.001). This is consistent with that reported in Snoek’s research [33]. Advanced supervision and technology, strong professional support and relatedness communication and feedback are the key advantages of digital health to enhance adherence. Digital health is an important medium for the delivery of exercise rehabilitation intervention, it not only integrates a variety of sensory systems to simulate movement situations, restores the realism and effectiveness of rehabilitation training, but also builds a social support network to support patients’ contact with physicians when concerns about rehabilitation arise after discharged [34,35]. However, in our study, exercise adherence was lower than expected, likely due to several contributing factors. One potential explanation is latency in the perceived benefits of exercise and a lack of self-efficacy. The participants might have higher expectations of recovery, but improvements in fitness levels gained through exercise are not immediate. When they failed to perceive recovery or encountered adverse symptoms, they chose to withdraw, affecting their persistence, enthusiasm, and adherence [36,37]. Self-efficacy is also an important determinant in exercise adherence [38]. And the socioeconomic and educational disadvantages among participants are negative to self-efficacy. Notably, 82 (82%) of the patients had an education level of junior high school or below, and 77 (77%) reported monthly incomes of less than US $840. These factors may have limited their understanding and engagement, consistent with existing literature that associates lower education and income with poor adherence [39-41]. In this trial, the stability of the connection between software and hardware products also needed to be improved and 2 patients had allergies to the hardware products, all of which need to be improved in the later studies.

#### Limitations

Our findings are limited by a short-term follow-up and the adherence was lower than expected. Further studies of the digital health program could use recruitment strategies aimed at extending the intervention period and follow-up, ensuring the effectiveness of rehabilitation programs, and maximizing the exercise adherence for patients’ physical fitness. Another limitation of this study is the absence of muscle mass measurement. Future research should incorporate muscle mass measurements to provide a more comprehensive assessment of the impact of exercise rehabilitation programs on physical fitness and overall health.

### Conclusion

The findings of this randomized controlled trial conducted in China suggested that novel digital intervention in exercise rehabilitation could improve exercise capacity and physical fitness among patients with PLC after hepatectomy. The intervention has the potential to increase access to rehabilitation treatment and reduce adverse symptoms in patients in the postsurgical recovery period.

## Supplementary material

10.2196/59228 Checklist 1 CONSORT-eHEALTH Checklist (V 1.6.1).

## Abbreviations

6MWT: 6-minute walk test
PLC: primary liver cancer
